# Supplementary material for: Shaping contactless radiation forces through anomalous acoustic scattering
Source: Nat Commun. 2022 Nov 1;13:6533. doi: 10.1038/s41467-022-34207-7 (PMC9626492; doi:10.1038/s41467-022-34207-7)
Supplement: Supplementary file 1 — Supplementary Information [file 41467_2022_34207_MOESM1_ESM.pdf]

Supplementary Information for

Shaping contactless radiation forces through anomalous acoustic scattering

M. Stein[1], S. Keller[1], Y. Luo[1], O. Ilic[1]*
[1]Department of Mechanical Engineering, University of Minnesota, Minneapolis, 55455, USA

Table of Contents

Experimental setup and the acoustic force balance 2

Metasurface force model and the unit cell design 4

Metasurface designs synthesized and fabricated in this work 5

Alternative metasurface topologies for shaping acoustic radiation forces 6

Analysis of the acoustic beam diameter for self-guiding........ 8

Experimental setup and the acoustic force balance

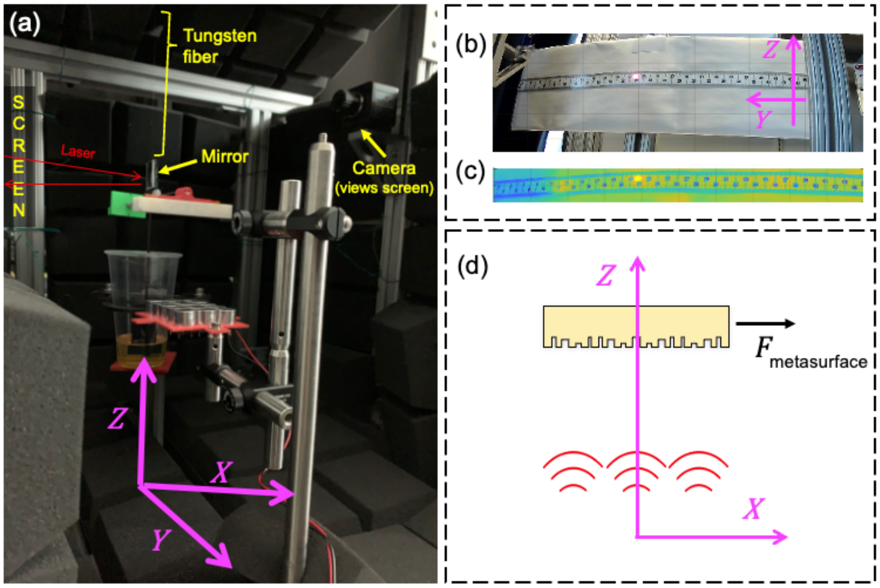

Figure S1. Experimental setup. a Photo of the experimental setup and the definition of the axes in the system (pink). The axis of rotation is the z-axis (gravity is also along z). Metasurface (white rectangular slab) and its counterweight (green block) are on the opposite ends of the pendulum rod (limited visibility from this angle). **b, c** Raw (**b**) and processed (**c**) camera image of the screen for real-time deflection tracking. The image is acquired by a horizontal-viewing camera on the opposite side of the screen (panel a, top right). Laser beam path is in the X-Y plane (horizontal plane). **d** X-Z plane schematic. The metasurface is facing down (-z), while the acoustic source faces up (+z).

Experimental arrangement and the relative orientation of the metasurface, the acoustic source, and the pendulum ensure the deflection measurement is a result of the metasurface force (as shown in Fig. S1a,d, and the figures in the main text):

- Z-axis is the pendulum rotation axis. Only a z-torque can lead to a deflection signal.
- Only the forces in the horizontal X-Y plane contribute to the z-torque. Forces along the z-axis are therefore not relevant.
- Metasurface unit cell profile varies in the X-direction, leading to a horizontal force $F_{\text{metasurface}}$ which contributes to the z-torque. This force is the focus of this work.
- Forces in the horizontal plane (but perpendicular to the metasurface variation along the X-direction), i.e., Y-forces, are not expected (theoretically, in simulation, or experiment), either from anomalous or conventional scattering. Even if such forces were present, the corresponding torque arm-length would be significantly shorter than for $F_{\text{metasurface}}$ (i.e., a fraction of the metasurface size ≪ the length of the pendulum rod), therefore minimizing their contribution to the z-torque.

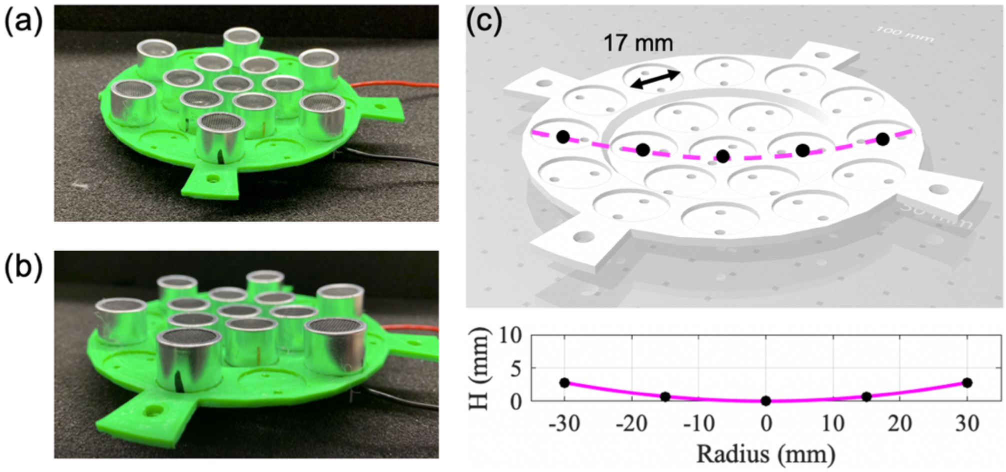

Figure S2. Holder for piezoelectric transducers that form the radiation source for the self-guiding experiment (Figure 3 of the main text, Supplementary Movie 3). **a**, **b** Photos of the holder. **c** CAD schematic of the holder and the plot showing the parabolic height variation ($H = br^2$, where $b = 3.125\ 10^{-3}\ mm^{-1}$).

Metasurface force model and the unit cell design

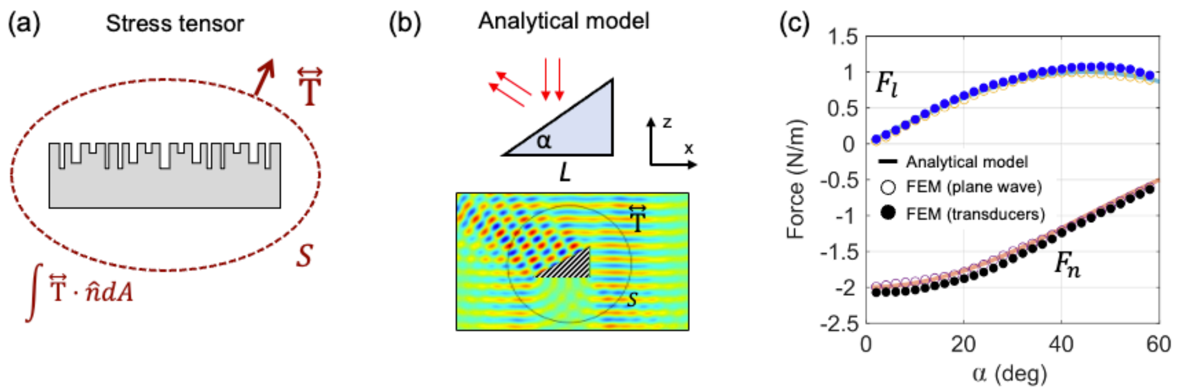

Figure S3. Stress tensor approach applied to a scattering metasurface. a Schematic. **b** Analytical model mimics a metasurface. **c** Comparing FEM simulation and the analytical model.

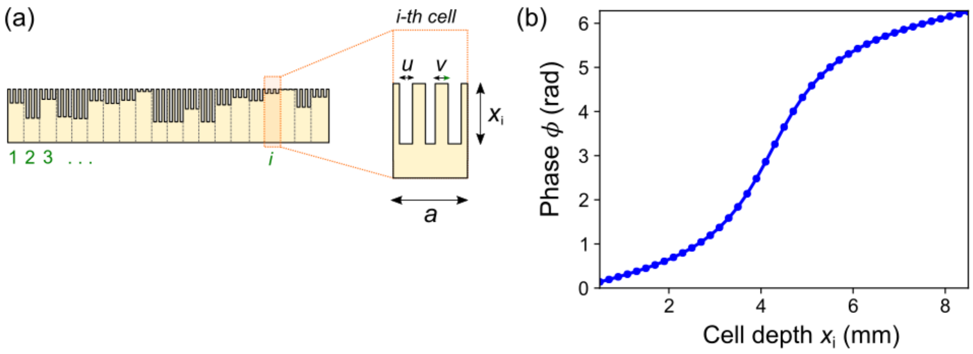

Figure S4. Metasurface unit cell. a Schematic of the metasurface and the building-block unit cell. **b** Reflected phase profile vs cell depth.

Metasurface designs synthesized and fabricated in this work

*All lengths are in mm

Metasurface 1 (Figure 2 of main text)

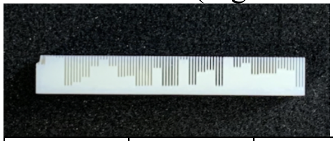

N = 1	2	3	4	5	6	7	8	9	10
2.56*	0.16	0.53	8.55	7.73	6.05	2.96	0.95	2.93	6.01
N = 11	12	13	14	15	16	17	18	19	20
5.69	8.55	8.48	3.42	8.55	8.54	0.87	8.17	4.50	3.90
N = 21	22	23	24	25	26	27	28	29	30
8.54	0.09	2.97	1.8	4.97	4.71	4.67	3.33	3.69	8.47

Metasurface 2 (Figure 3 of main text)

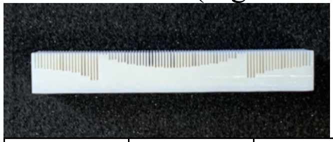

N = 1	2	3	4	5	6	7	8	9	10
4.23*	4.48	4.75	5.07	5.53	6.24	7.44	0.56	1.95	2.77
N = 11	12	13	14	15	16	17	18	19	20
3.28	3.64	3.92	4.16	4.40	4.40	4.16	3.92	3.64	3.28
N = 21	22	23	24	25	26	27	28	29	30
2.77	1.95	0.56	7.44	6.24	5.53	5.07	4.75	4.48	4.23

Metasurface 3 (Figure 4 of main text)

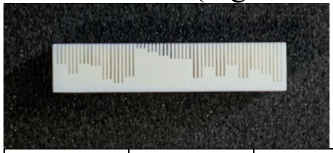

N = 1	2	3	4	5	6	7	8	9	10
4.30*	6.74	4.35	4.99	8.14	9.00	7.58	1.14	2.03	2.85
N = 11	12	13	14	15	16	17	18	19	20
3.53	3.64	7.96	5.42	7.62	5.10	8.03	6.09	7.28	9.00

Alternative metasurface topologies for shaping acoustic radiation forces

The acousto-mechanical metasurface physics that we report is general and not unique to a particular unit cell design. As a demonstration, in addition to the unit cell profile depicted in the main text and Fig. S4, we investigate a metasurface composed of a coiled unit cell topology. Figure S5a shows an example implementation where a unit cell consists of five horizontal arms (three on the left side and two on the right side). For operation at the target frequency of 20 kHz, we chose the following fixed parameters $d = 0.25$ mm, $a = 2.14$ mm, $H = 2.14$ mm, and the wall thickness t is the variable parameter. Figure S5b shows the relative phase variation of the reflected wave as t is varied.

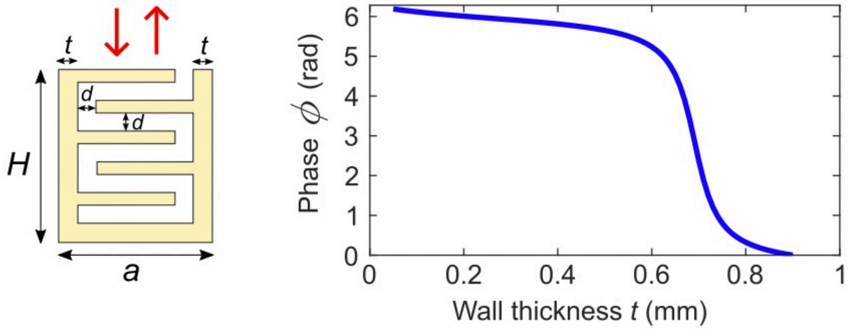

Figure S5. Example alternative unit cell. a Schematic of an alternative metasurface profile based on a space-coiled unit cell. **b** Reflected phase profile vs wall thickness *t*.

Following the same design process outlined in the Methods section, we arrive at the analytical and the refined/optimized metasurface 1 with the space-coiled unit cell profile. Because the unit cell width a is smaller than before, we increased the number of unit cells to N=36 to maintain the same overall size of the metasurface. As in the case of the groove-like metasurface of the main text, the optimization step for the space-coiled design resulted in a substantial lateral acoustic force enhancement (1.39x increase). The analytical designs of the two unit cell topologies—groove and space-coiled—otherwise show comparable performance (i.e., the optimized coiled metasurface has a 1.24x stronger lateral force relative to the optimized grooved metasurface). The coiled design has a smaller minimal feature size as well as a region of sensitive phase vs wall thickness relationship (steep slope around t~0.7 mm in Fig. S5b) which could pose potential fabrication challenges. The schematics of coiled metasurfaces and the tabulated values for the unit cell wall thicknesses (t) are presented below.

Coiled metasurface (analytical; all lengths are in mm)

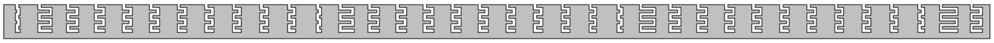

N = 1	2	3	4	5	6	7	8	9	10
0.90	0.51	0.62	0.65	0.67	0.68	0.69	0.70	0.71	0.73
N = 11	12	13	14	15	16	17	18	19	20
0.76	0.86	0.47	0.61	0.65	0.67	0.68	0.69	0.70	0.71
N = 21	22	23	24	25	26	27	28	29	30

0.73	0.76	0.84	0.41	0.60	0.64	0.66	0.68	0.69	0.70
N = 31	32	33	34	35	36				
0.71	0.73	0.75	0.82	0.34	0.59				

Coiled metasurface (optimized; all lengths are in mm)

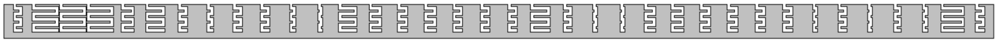

N = 1	2	3	4	5	6	7	8	9	10
0.71	0.05	0.05	0.05	0.56	0.31	0.70	0.83	0.71	0.71
N = 11	12	13	14	15	16	17	18	19	20
0.82	0.90	0.34	0.59	0.67	0.59	0.68	0.69	0.70	0.30
N = 21	22	23	24	25	26	27	28	29	30
0.73	0.90	0.90	0.63	0.57	0.65	0.66	0.68	0.68	0.86
N = 31	32	33	34	35	36				
0.71	0.90	0.76	0.85	0.14	0.69				

Analysis of the acoustic beam diameter for self-guiding

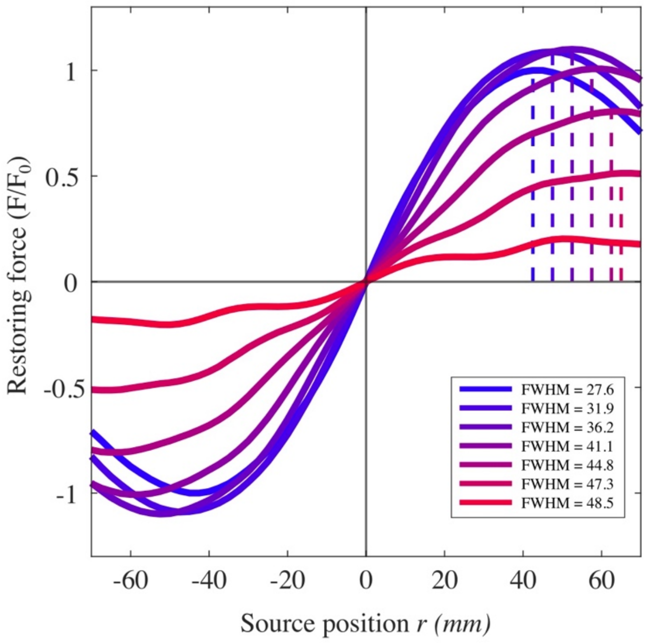

Figure S6. Relationship between the beam diameter and the self-guiding force for the metasurface 2 of the main text (Figure 3). For each beam diameter (indicated by the full-width half-maximum value in mm), the force is numerically evaluated for a range of source positions (relative displacements between the metasurface center and the beam axis – see schematic in Figure 3b of main text). Forces are normalized to the peak force (F_0) of the narrowest/first analyzed beam width. Initially, increasing the beam diameter helps enhance the peak restoring acoustic radiation force. However, as the beam diameter becomes broader, the countering contribution from the opposing metasurface symmetric side becomes stronger, and the overall self-guiding force is weakened. This implies that the relative source displacement for which the force is the strongest (for a given diameter) would increase as the beam diameter increases, and this trend is indeed observed (i.e., the vertical dashed lines shift from left to right).
