# Supplementary material for: Shaping contactless radiation forces through anomalous acoustic scattering
Source: Nat Commun. 2022 Nov 1;13:6533. doi: 10.1038/s41467-022-34207-7 (PMC9626492; doi:10.1038/s41467-022-34207-7)
Supplement: Supplementary file 3 — Description of Additional Supplementary Files [file 41467_2022_34207_MOESM3_ESM.docx]

**Description of Additional Supplementary Files**

**Title:** Supplementary Movie 1

**Description:** Metasurface motion resulting from tailored anomalous acoustic scattering (companion to Figure 2). The source is turned on ~3 sec after the start of the movie. The playback rate is 10x of the original camera video. The acoustic source is inaudible (20 kHz). In this actuation configuration, the metasurface is visible from the top view.

**Title:** Supplementary Movie 2

**Description:** Metasurface motion resulting from tailored anomalous acoustic scattering (companion to Figure 2). The source is turned on ~3 sec after the start of the movie. The playback rate is 10x of the original camera video. The acoustic source is inaudible (20 kHz). Top view is shown – the metasurface features face the transducers and are not visible from this angle. This configuration is used in Fig. 3 and Fig. 4, as it more explicitly highlights the contribution of metasurface-induced forces. See Figure S1.

**Title:** Supplementary Movie 3

**Description:** Self-guiding metasurface motion (companion to Figure 3). The metasurface autonomously tracks and guides itself to follow the movement of the acoustic source. The playback rate is 100x of the original camera video. The acoustic source is inaudible (20 kHz). Top view is shown – metasurface features face the transducers and are not visible from this angle. See Figure S1.
